# The influence of distance and quality on utilisation of birthing services at health facilities in Eastern Region, Ghana

**DOI:** 10.1136/bmjgh-2019-002020

**Published:** 2020-02-10

**Authors:** Winfred Dotse-Gborgbortsi, Duah Dwomoh, Victor Alegana, Allan Hill, Andrew J Tatem, Jim Wright

**Affiliations:** 1 School of Geography and Environmental Science, University of Southampton, Southampton, UK; 2 WorldPop Research Group, School of Geography and Environmental Science, University of Southampton, Southampton, UK; 3 Department of Biostatistics, School of Public Health, College of Health Sciences, University of Ghana, Legon, Accra, Ghana; 4 Population Health Unit, Kenya Medical Research Institute - Wellcome Trust Research Programme P.O. Box 43640-00100, Nairobi, Kenya; 5 Faculty of Science and Technology, Lancaster University, Lancaster, UK; 6 Social Statistics and Demography, University of Southampton, Southampton, UK

**Keywords:** maternal health, geographic information systems, obstetrics, public health, epidemiology

## Abstract

**Objectives:**

Skilled birth attendance is the single most important intervention to reduce maternal mortality. However, studies have not used routinely collected health service birth data at named health facilities to understand the influence of distance and quality of care on childbirth service utilisation. Thus, this paper aims to quantify the influence of distance and quality of healthcare on utilisation of birthing services using routine health data in Eastern Region, Ghana.

**Methods:**

We used a spatial interaction model (a model that predicts movement from one place to another) drawing on routine birth data, emergency obstetric care surveys, gridded estimates of number of pregnancies and health facility location. We compared travel distances by sociodemographic characteristics and mapped movement patterns.

**Results:**

A kilometre increase in distance significantly reduced the prevalence rate of the number of women giving birth in health facilities by 6.7%. Although quality care increased the number of women giving birth in health facilities, its association was insignificant. Women travelled further than expected to give birth at facilities, on average journeying 4.7 km beyond the nearest facility with a recorded birth. Women in rural areas travelled 4 km more than urban women to reach a hospital. We also observed that 56% of women bypassed the nearest hospital to their community.

**Conclusion:**

This analysis provides substantial opportunities for health planners and managers to understand further patterns of skilled birth service utilisation, and demonstrates the value of routine health data. Also, it provides evidence-based information for improving maternal health service provision by targeting specific communities and health facilities.

**Key questions**

**What is already known?**

- Skilled attendance at birth is a key intervention in reducing maternal mortality.
- Skilled attendants generally supervise births at health facilities in Ghana.
- Cross-sectional studies using household surveys have found both distance and quality of care affect skilled attendance at birth.

**What are the new findings?**

- Distance travelled had a more profound influence on health facility births than quality of care.
- Most women travelled beyond their nearest health facility to give birth, sometimes crossing regional boundaries to do so.

**What do the new findings imply?**

- Access to skilled birth should be improved by placing health facilities closer to communities and improving the quality of care at existing health facilities.
- We can further understand maternal health utilisation through routine health management information systems data analysis.

## Introduction

Although the maternal mortality ratio fell by 37% globally between 2000 and 2015,^1^ sub-Saharan Africa still records 546 deaths per 100 000 live births.^2^ While the global maternal mortality ratio decreased by 1.5% per year from 1990 to 2015, in sub-Saharan Africa it declined at half this rate.^3^ Skilled attendance at childbirth has been identified as the key intervention for the reduction of maternal mortality.^4^ When births occur outside health facilities in low and middle-income countries, they are less likely to have a skilled attendant present.^5^ Previously, a skilled attendant was defined as a qualified health professional such as a midwife, doctor or nurse specially trained with skills to manage pregnancy, supervise births and care immediately after birth.^6^ An updated definition of a skilled birth attendant has a broader team including nurses, midwives, anaesthetists and specialised doctors such as obstetricians and paediatricians.^7^ Instead of emphasising job roles, the updated definition of a skilled birth attendant focuses on actual professional competencies alongside relevant training of personnel, together with an enabling working environment.

When a woman goes into labour or when obstetric complications occur, reaching the nearest well-equipped health facility in the shortest possible time is essential for the prevention of maternal mortality. The decision to use facility-based maternal health services is affected by the geographical distribution of health facilities and by the distance to such facilities.^8^ There is evidence to support a link between distance to facility and health outcomes.^9^ In addition, the effects of facility services and infrastructure on attendance are examined here. Quality is assessed using measures of human resources, infrastructure, equipment, medicines and supplies.^10^

Few studies have assessed the influence of both distance and the quality of healthcare on the way skilled childbirth attendance services are used in low and middle-income countries.^11 12^ These studies have used data from specific research projects such as clinical trials^11^ or Demographic and Health Surveys (DHS),^13 14^ rather than being based on routinely collected health management information systems (HMIS) data. Studies using the DHS have examined attendance at any health facility by distance to the nearest facility, but have not examined attendance at specific, named facilities nor the impact of quality of maternal care services provided on health facility use.^15^ The DHS and similar surveys (eg, multiple indicator cluster survey) do not record facility names and such analyses are further complicated by the random displacement of household cluster locations for data protection purposes.^16^

Therefore, the aim of this study was to use routinely collected childbirth data from an HMIS for hospitals in Eastern Region, Ghana, to determine the influence of distance and the quality of healthcare on the use of birthing services. In addition, we analysed the characteristics of women who bypassed their nearest health facility in order to attend an alternative centre. The study thus seeks to develop methods for making fuller use of routinely collected childbirth data for policy and interventions. To our knowledge, the study also produced the first visualisation of childbirth-related patient flows to facilities at a subnational level in Africa.

## Methods

A cross-sectional study design was used to examine the number of women using public secondary healthcare facilities among women expected to give birth in the Eastern Region of Ghana from January to December 2016. Less than 1% of births occurring in Eastern Region’s health facilities do not have a skilled attendant, while conversely, skilled attendance of home births in the region is very rare. Our study therefore uses birthing at a facility as a proxy for skilled birth attendance.^17^

### Selection of sites

The Eastern Region was selected because there were spatial data on location of health facilities and communities. In addition, since HMIS coverage of private health facilities is low, the Eastern Region was chosen because its healthcare facilities are largely publicly funded. Finally, the study focused on childbirth in hospitals only, because of the lack of electronic birth records in primary healthcare facilities.

### Data

The study used four secondary data sets, namely HMIS records of hospital-based births; locations of health facilities reporting through the HMIS; a nationally representative sample survey of emergency obstetric and newborn care (EmONC) services at health facilities; and gridded estimated number of pregnancies.

The HMIS data were extracted from Ghana’s District Health Information Management Systems 2 (DHIMS2)^18^ database. DHIMS2 is a database for storing routine health data from patient interactions at health facilities in Ghana. They comprised the individual-level records of 26 563 routine births at 19 public hospitals in the Eastern Region, Ghana, for 2016. In addition to the facility of birth, DHIMS2 recorded mother’s place of residence, age, occupation, educational status, health insurance, parity, type of birth and birth outcome. Alongside these individual-level data, DHIMS2 records separately the monthly aggregate number of women giving birth in health facilities also used here.

Healthcare quality at these facilities was assessed based on a nationally representative sample survey of EmONC services at 124 health facilities in Eastern Region (including 22 hospitals) in 2010.^19^ In the Eastern Region, health facilities that recorded at least five births each month in 2009 were included in the EmONC survey. EmONC data on just two hospitals (Seventh-day Adventist and St Joseph) were not available. The EmONC assessment collected data on infrastructure, human resources, drugs, equipment, supplies, signal functions and other essential maternal and newborn health variables to quantify indicators that reflect the state of EmONC as recommended by the WHO.^20^

The spatial distribution of potential demand for obstetric care was quantified using a gridded (100×100 m) map layer of estimated pregnancy for 2015.^21 22^ The WorldPop programme generated these estimates by redistributing census-derived number of pregnancies for electoral areas down to grid cells based on land cover and settlement extents derived from satellite imagery.

**Figure 1:**
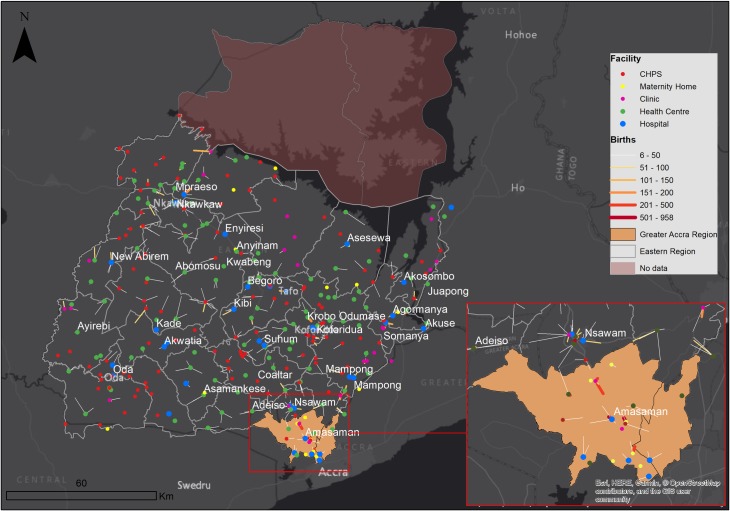
Flows from communities to health facilities; expected flow of women determined by assigning each woman to the nearest health facility that reported a birth; flow line width and colour intensity depicts frequency of flows whereas length shows distance. CHPS, community health planning and services.

A 5 km buffer zone was created around the Eastern Region to partially account for usage of the region’s healthcare facilities by residents of neighbouring regions. Following preliminary exploration of HMIS data, the Ga East and West districts from the Greater Accra region (figure 1) were added to the model due to the large patient inflows from these districts. The demand for services from communities in the buffer zone was included but not their health facilities, since we had no access to the data on births in the neighbouring regions.

**Figure 4:**
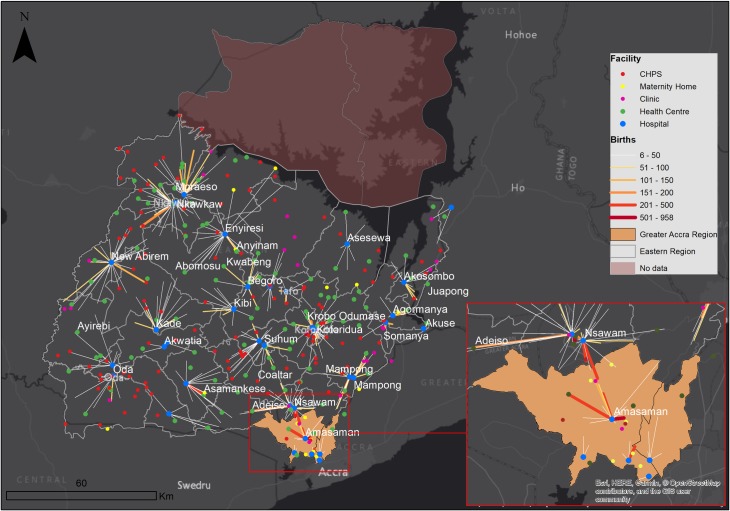
Flows from communities to health facilities; expected flow of women determined by assigning each woman to the nearest hospital; flow line width and colour intensity depicts frequency of flows whereas length shows distance. CHPS, community health planning and services.

**Figure 5:**
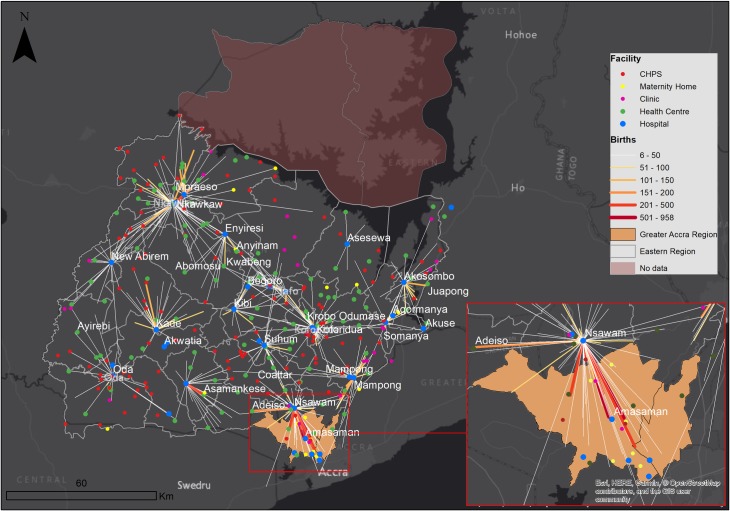
Flows from communities to health facilities; observed flow of women to hospitals; flow line width and colour intensity depicts frequency of flows whereas length shows distance. CHPS, community health planning and services.

Rural/urban analysis of results was achieved by overlaying a map layer of mothers’ places of residence on an urban extent map layer derived from satellite imagery.^23^

### Locating mothers’ places of residence

Geocoding, a technique for converting place names to map coordinates, was used to locate mothers’ places of residence for subsequent mapping and analysis. Mothers’ places of residence in HMIS records were geocoded by manually matching settlement names where women lived with locational data sets from the Centre for Geographical Information Systems and Remote Sensing-University of Ghana, Google Maps and a Global Positioning System survey of settlements conducted by the Ghana Health Services. There were no differences in sociodemographic characteristics between women whose place of residence was successfully geocoded and those without location information (see online supplementary appendix A).

10.1136/bmjgh-2019-002020.supp1 Supplementary data

### Individual-level analysis of facility attendance for childbirth

The completeness of individual-level records at hospitals was assessed by comparing individual and aggregate numbers of women in DHIMS2. Since straight-line distance has been shown to be highly correlated with more sophisticated distance metrics, such as drive times, in settings without significant physical barriers to movement,^24^ straight-line distance was calculated between each mother’s geocoded community of residence and the hospital where the childbirth took place. For comparison with these observed distances travelled to give birth, two expected distances travelled were calculated, assuming that facility choice was affected only by geography. The first expected distance was calculated to the nearest hospital from each mother’s community of residence, while the second was calculated to the nearest primary or secondary healthcare facility that had recorded a birth. For the expected travel to any facility (primary or secondary) providing birthing services, 323 health facilities comprising 134 (41%) community health planning and services, 26 (8%) clinics, 110 (34%) health centres, 37 (11%) hospitals and 16 (5%) maternity homes were used as destination health facilities for the second set of expected distances, since they recorded births. Then, a non-parametric Kruskal-Wallis test was used to compare the differences in distance (observed minus expected) by sociodemographic characteristics.

### Population-level analysis and flow visualisation of facility attendance for childbirth

To visualise the mothers’ travel from place of residence to destination health facility, three maps were developed, two illustrating expected flows of mothers to facilities and the other showing observed flows. The first expected travel was to any health facility providing birthing services, and the second one to hospitals only. Patient flow lines linking communities and facilities were generated in QGIS (V.3.4) and mapped in ArcGIS (V.10.5). Flows less than five women were excluded to improve clarity.^25^

### Spatial interaction modelling

A spatial interaction model predicts the flow of people between an origin and a destination based on destination attractiveness, demand for services at the origin and distance between origin and destination. This study implemented a spatial interaction model to predict recorded patient flows from communities to facilities, since such models quantify how distance affects healthcare facility attendance and provide goodness-of-fit diagnostics of model performance.^26–28^ The number of women travelling from a given origin community to a given hospital was modelled as a function of the number of pregnancies in the community the woman came from, healthcare quality at the destination hospital, the number of beds as a proxy for hospital size and the distance between the community and hospital, expressed as an exponential distance decay function.

A summary quality of healthcare index was created from the EmONC facility survey data. This bespoke quality index was based on six categories of variables representing physical size, availability of medicines, medical and non-medical supplies, infection prevention and EmONC signal functions. The categories were selected based on Hulton and colleagues’ framework for assessing quality of maternal care using two dimensions, namely experience and provision of care.^29^ The categories were range standardised and weighted to achieve a summary index. Higher weights were assigned to those categories (physical size and non-medical supplies) more apparent to women giving birth. The variables in each category and weights are provided in online supplementary appendix B.

To estimate the demand for maternal healthcare, estimated pregnancies from the WorldPop gridded map layer were summed for zones representing women’s communities of residence. To control for varying incompleteness of individual-level records per facility, the ratio of individual-level to aggregate reported births (from HMIS) was also included in the model.

Four different models (Poisson, negative binomial, zero-inflated Poisson and zero-inflated negative binomial models) were fitted to quantify the effect of the predictors on number of facility births (table 1). The performance of these models was evaluated based on the Akaike information criterion.

**Table 1:** Results from the spatial interaction model predicting births in 19 hospitals in Eastern Region, Ghana, in 2016

Predictors	Prevalence ratio (95% CI)	P value
Distance (km)	0.935 (0.930, 0.940)	<0.001
Per cent quality score	1.01 (0.999, 1.02)	0.069
Number of estimated pregnancies	1.002 (1.001, 1.002)	<0.001
Per cent completeness of birth data	1.02 (1.016, 1.024)	<0.001
Number of inpatient beds	1.002 (1.001, 1.004)	0.001
Model inflation distance	1.08 (1.074, 1.087)	<0.001

### Patient and public involvement

This research was done without patient involvement. Patients were not invited to comment on the study design and were not consulted to develop patient-relevant outcomes or interpret the results. Patients were not invited to contribute to the writing or editing of this document for readability or accuracy.

## Results

### Individual-level analysis of facility attendance for childbirth

**Figure 2:**
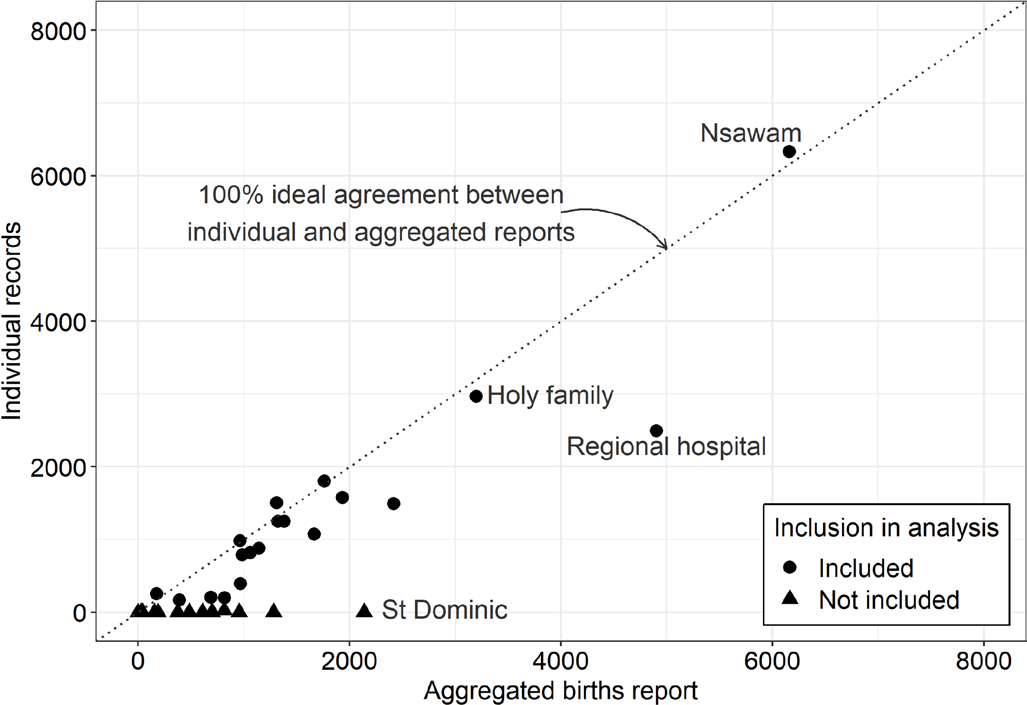
Completeness of health management information systems (HMIS) individual birth records for 35 hospitals in Eastern Region, Ghana, during 2016. Hospitals below the dotted line returned less individual records compared with aggregates and more than 100% individual records in hospitals above the line.

Figure 2 shows the proportion of individual records documented via the HMIS for 35 secondary care facilities, compared with the number of women documented in aggregate routine reports. The 26 563 individual records received were incomplete, comprising only 63.4% of aggregate reported number of women giving birth at hospitals in 2016. Individual-level records were almost incomplete or totally non-existent in 16 out of 35 hospital facilities, whereas individual records exceeded aggregate number of women in five hospitals (above dotted line in figure 2). Most of the health facilities without individual records were either private health facilities or faith-based hospitals and returned fewer aggregate birth counts. The 16 hospitals with incomplete records plus two hospitals (Koforidua Seventh-day Adventist and St Joseph) lacking EmONC service quality data were excluded from subsequent population analysis, leaving 17 hospitals (see online supplementary appendix C for flow diagram on excluded data).

Although there were non-identifiable, ambiguous or unavailable locations for some patients, the places of residence of 23 246 (87.5%) women were successfully geocoded to communities of origin and after further cleaning, 21 856 (82.3%) were included in the individual-level analysis. Table 2 summarises the characteristics of these 21 856 women who gave birth in health facilities. More women (81.9%) were between 20 and 40 years and resided mainly in rural areas. The majority were educated up to junior high school level (56.2%) and were either trading or farming (42.6%), whereas 5.6% were still in school. Almost all women had health insurance (99%), but there were 100 more uninsured women in rural dwellings relative to urban settings. Most women gave birth by spontaneous vaginal births and were discharged without complications. Generally, women travelled 5.73 km to a hospital, but rural women travelled farther (7.53 km) than urban women (1.04 km).

**Table 2:** Rural/urban characteristics of women and summary of distance travelled (n=21 856)

Characteristics	Rural (%)	Urban (%)	Total (%)
Age (years) (n=21 856)			
10–20	2710 (16.5)	841 (15.6)	3551 (16.2)
20–30	8653 (52.6)	2901 (53.6)	11 554 (52.9)
30–40	4784 (29.1)	1550 (28.7)	6334 (29.0)
40–50	301 (1.8)	116 (2.1)	417 (1.9)
Education (n=21 289)			
No formal education	2332 (14.6)	655 (12.3)	2987 (14.0)
Primary	1118 (7.0)	937 (17.6)	2055 (9.7)
Junior high	9018 (56.5)	2954 (55.5)	11 972 (56.2)
Senior high	2495 (15.6)	452 (8.5)	2947 (13.8)
Tertiary	1008 (6.3)	320 (6.0)	1328 (6.2)
Occupation (n=21 856)			
Employed	3878 (23.6)	1124 (20.8)	5002 (22.9)
Trader/farmer	6815 (41.4)	2496 (46.2)	9311 (42.6)
Student	954 (5.8)	270 (5.0)	1224 (5.6)
Unemployed	1958 (11.9)	584 (10.8)	2542 (11.6)
Unspecified	231 (1.4)	138 (2.6)	369 (1.7)
Others	2612 (15.9)	796 (14.7)	3408 (15.6)
Health insurance (n=21 856)			
Non-insured	158 (1.0)	51 (0.9)	209 (1.0)
Insured	16 290 (99.0)	5357 (99.1)	21 647 (99.0)
Outcome of birth (n=21 856)			
Absconded	5 (0.0)	5 (0.1)	10 (0.0)
Died	4 (0.0)	0 (0.0)	4 (0.0)
Discharged	16 424 (99.9)	5401 (99.9)	21 825 (99.9)
Transferred	13 (0.1)	2 (0.0)	15 (0.1)
Unspecified	2 (0.0)	0 (0.0)	2 (0.0)
Type of birth (n=21 856)			
Normal births and normal births with episiotomy	12 625 (76.8)	4051 (74.9)	16 676 (76.3)
Caesarean section and other surgical procedures	3823 (23.2)	1357 (25.1)	5180 (23.7)
Parity (n=21 119)			
Never given birth	3817 (24.0)	1185 (22.6)	5002 (23.7)
1–3	10 081 (63.5)	3499 (66.7)	13 580 (64.3)
4 or more	1978 (12.5)	559 (10.7)	2537 (12.0)
Distance travelled (km)			
Mean (SE)	8.41 (0.05)	4.44 (0.09)	7.47 (0.05)
Median (IQR)	7.53 (9.21)	1.04 (4.49)	5.73 (10.59)

The majority of women bypassed the nearest hospital (56%) or the nearest health facility (76%). More than half of the women bypassed both the nearest hospital and primary health facility (54.2%). Bypassing the nearest primary health facility was more prevalent than among hospitals. Table 3 reflects the way mothers bypass secondary and primary health facilities providing birthing services by comparing the observed travel to expected distances at the health facility tiers. The median expected travel distances to secondary and primary facilities were 3.3 km (IQR: 7.1) and 1 km (IQR: 2.7), respectively, but the observed was 5.7 km (IQR: 10.6). This shows that women were travelling longer distances and bypassing closer health facilities. Older women travelled longer distances than expected to hospitals (p<0.001). Education, occupation and parity had significant differences among the various groups of women in travel to both tiers of health facilities (p<0.001). In contrast, there were no significant differences for health insurance, outcome of birth and whether a woman had a caesarean section or not.

**Table 3:** Differences in distance travelled calculated by subtracting expected distance to primary and secondary health facilities from the observed distances (n=21 856)

	O: Observed distance travelled (km)		E1: Expected distance to nearest secondary care (km)		E2: Expected distance to nearest facility recording births (km)		Test statistic (for difference in O-E1 by patient characteristic)	Test statistic (for difference in O-E2 by patient characteristic)
	Median	IQR	Median	IQR	Median	IQR	Kruskal-Wallis	Kruskal-Wallis
Age (years)							<0.001	0.66
10–20	6.31	10.11	4.04	7.85	0.99	2.64		
20–30	5.46	10.31	3.16	6.90	0.97	2.71		
30–40	5.77	11.06	3.16	6.66	0.94	2.84		
40–50	6.22	10.49	3.79	7.93	0.93	2.77		
Education							<0.001	<0.001
No formal education	5.59	6.59	3.95	6.25	0.84	0.98		
Primary	3.19	9.23	1.84	8.23	0.84	0.85		
Junior high	6.22	11.02	3.16	6.91	1.07	3.29		
Senior high	5.54	8.50	3.16	5.97	1.20	2.84		
Tertiary	4.41	9.18	1.83	5.84	0.72	1.50		
Occupation							<0.001	<0.001
Employed	5.72	11.15	2.12	5.61	1.05	3.05		
Trader/farmer	5.62	11.06	3.16	6.95	0.98	2.94		
Student	5.54	8.50	3.63	6.70	1.00	2.31		
Unemployed	6.22	10.42	3.63	7.00	1.09	2.84		
Unspecified	8.79	15.07	6.02	11.93	0.60	2.91		
Others	5.50	7.22	3.97	6.92	0.84	0.96		
Health insurance							0.83	0.40
Non-insured	4.82	12.43	3.16	8.38	0.98	2.78		
Insured	5.73	10.53	3.34	7.08	0.97	2.73		
Outcome of birth							0.04	0.46
Absconded	2.95	3.97	0.84	3.00	0.84	0.86		
Died	1.70	19.93	1.69	17.56	0.50	1.52		
Discharged	5.73	10.53	3.34	7.11	0.97	2.73		
Transferred	7.98	12.38	5.45	9.38	0.64	0.82		
Unspecified	14.98	1.82	14.98	1.82	1.51	1.06		
Type of birth							0.84	0.18
Normal births and normal births with episiotomy	5.75	10.01	3.39	7.08	0.98	2.85		
Caesarean section and other surgical procedures	5.62	11.15	3.16	7.13	0.84	2.30		
Parity							<0.001	<0.001
Never given birth	6.02	9.72	3.21	7.13	0.89	2.66		
1–3	5.45	10.31	3.16	6.67	0.96	2.64		
4 or more	6.49	10.68	3.75	7.37	1.05	2.99		

### Population-level analysis and flow visualisation of facility attendance for childbirth

**Figure 3:**
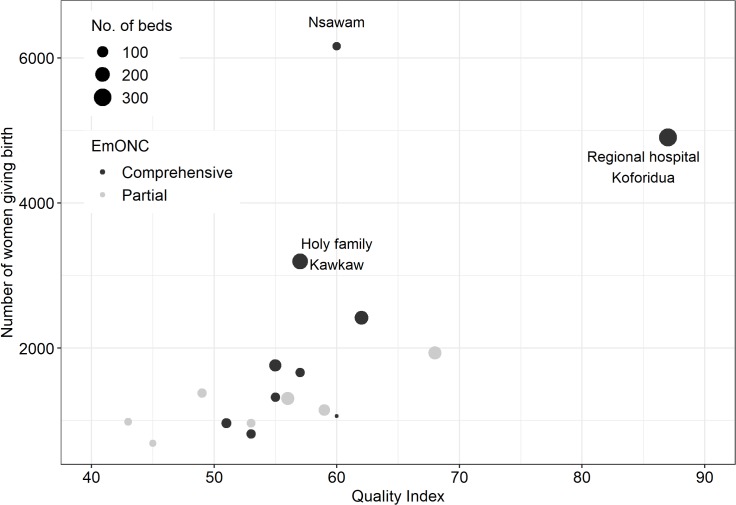
Number of women giving birth in health facilities in 2016 versus quality index, available beds and emergency obstetric and newborn care (EmONC) status for 17 hospitals in Eastern Region, Ghana (excludes Seventh-day Adventist and St Joseph).

Figure 3 highlights the relationship between births and healthcare quality. The derived quality index ranged from 43% to 87%. The Eastern regional hospital had the highest quality. Most hospitals with higher number of women had comprehensive EmONC services, whereas all hospitals with partial EmONC services recorded less than 2000 women. Although Nsawam Hospital had a low bed complement (proxy for physical size), it had the largest number of women giving birth. There was more variability among comprehensive facilities compared with the partially designated hospitals.

There is a clear difference in utilisation patterns, as shown in figures 1, 4 and 5. Women would have travelled shorter distances if they had attended the nearest health facility or nearest hospital. The observed journeys show that women travelled farther than expected to use a birthing service. As expected, most of the larger flows were closer to the hospitals. The Regional, Nsawam and Holy Family Hospitals located at Koforidua, Nsawam and Nkawkaw, respectively, stand out as the most used, irrespective of distanceThere were large flows from Greater Accra region to Nsawam Hospital in the Eastern Region.

The results from the zero-inflated negative binomial model showed that, for each kilometre increase in distance from a health facility, the prevalence ratio of the number of women giving birth in a health facility between origin and destination pairs decreases by 6.7% (95% CI 6 to 7; p<0.001). The inflate coefficient for distance suggests that for each unit increase in distance from facility, the prevalence rate of an inflated zero increases by 8% (95% CI 7.4 to 8.4; p<0.001). For each additional expected pregnancy, the prevalence ratio of the number of women giving birth in a health facility increases by 0.2% (95% CI 0.1 to 0.2; p<0.001). Unexpectedly, quality of care was marginally insignificant (p=0.069). The effect of other facility birth predictors can be found in table 1. Other model results from initial exploration are presented as online supplementary appendix D.

**Figure 6:**
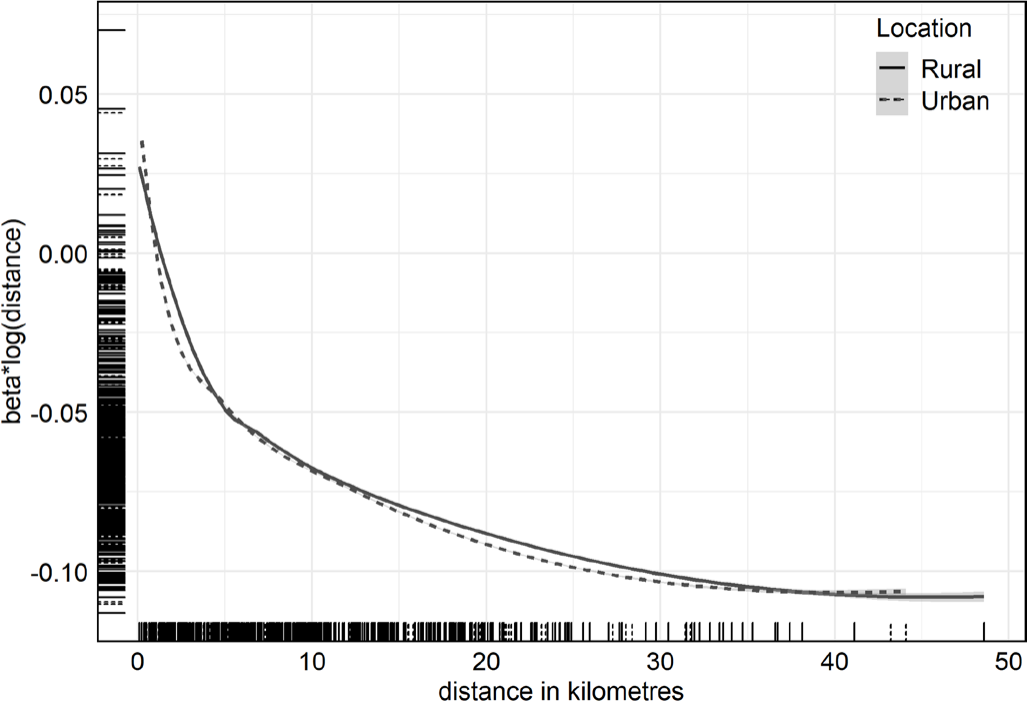
Effect of distance to facility on the number of women using birthing services based on a zero-inflated negative binomial regression model. The short lines on the axes show the distribution of origin–destination points along the lines.

The effect of distance on skilled birth service utilisation was assessed using the modelled distance decay curve in figure 6. The exponential effect of distance on skilled births is seen as the number of women decreased rapidly within the first 10 km and a less steep slope thereafter. There was a more rapid decrease in distance for urban women compared with their rural counterparts. The slopes for both groups were similar between 5 and 12 km, then after that urban flows decreased marginally faster. The rug plot indicates fewer travels beyond 25 km.

## Discussion

This paper presents the first geovisualisation of skilled birth interaction between named hospitals, and communities, using routine HMIS data. The flow visualisation reveals the previously unknown true catchment extent of some hospitals and provides significant opportunities to improve accessibility to quality maternal healthcare services. Likewise, health managers can plan and implement maternal health interventions to meet observed demand beyond administrative boundaries. As HMIS data availability, completeness and quality improve, data sets such as the one used in this study should enable regularly updated analyses of geographic patterns of birthing service utilisation. Further investment in health information management to address challenges such as incompleteness of records could support precise decision-making^30^ through greater reliance on routinely collected data and a lower need for makeshift, externally funded projects.

The relationship found between distance and health facility births within secondary care supports existing literature on the negative influence of distance on skilled childbirth attendance in low and middle-income countries.^11–14 31^ Distance significantly reduced the prevalence of mothers giving birth in a hospital by 6.7% per km increase, an effect comparable to the 4.4% found in rural India.^31^ Our finding is however lower than the 29% reduction in facility-based births reported in Zambia^12^ when distance to the facility doubled. The odds of delivery decreased by 61% per km increase in the log of distance, which is broadly comparable to a nationwide study in Ghana that reported 54%.^14^ However, it was higher than the 38% found in the Brong Ahafo region of Ghana.^11^ All of these other studies used survey data, analysed individual events and controlled for their individual determinants using logistic models that specified childbirth outside a health facility as the reference category. Some studies also log transformed their distance measures. In contrast, this study used origin–destination pairs in a spatial interaction model, so the different methods applied could account for some of the apparent differences in observed distance decay relative to other studies. Furthermore, the effect of distance on rural/urban women shown in figure 6 did not show much difference within the first 10 km as expected because the spatial data used to classify rural/urban settlements captured most periurban areas as rural.

Quality of care did not significantly increase the number of women giving birth in health facilities, comparable to related studies in other sub-Saharan African countries.^11 13^ Since our choice of indicators for the healthcare quality of care in this study shares similarities with a study in rural Zambia, in which quality of care was significant,^12^ it was surprising we did not observe such an association. Our range of indicators for the quality of care index reflected characteristics more apparent to women, a feature that is essential for observing an association between quality of care and increased skilled births.^13^ The study in rural Zambia on the influence of distance and level of care on place of birth included variables such as availability of water, privacy, electricity, waiting area for family members and functioning patient toilets in addition to other measures such as drugs, level of care, human resource and physical size in the summary index.^12^

The difference between expected and observed flows could be explained by the influence of distance and quality of care, which makes women prone to bypassing the nearest hospital to use another. Further exploration revealed that 76% of the women bypassed the nearest health facility (primary or secondary) providing birthing services, similar to the 75.4% found bypassing the nearest facility in Tanzania, which was explained by quality of care.^32^ However, rates of bypassing hospitals only were approximately 20% lower. Other reasons that could account for bypassing of health facilities are previous experience in a facility, which influences quality, access to services and mistrust.^33 34^ Bypassing is due overwhelmingly to patient choice, since only 18 cases were transferred between hospitals by health staff. Further studies are needed to find the reasons for bypassing in the region.

There was a high inter-regional flow of patients from the Ga East and West municipalities in Greater Accra region to use birthing services at Nsawam Hospital in Eastern Region. Although the nearest hospitals to these areas of Greater Accra in terms of straight-line distance lay within the same region, an examination of drive times within Google Maps suggested that drive times from these neighbourhoods to Nsawam Hospital and hospitals within Greater Accra were similar. This inter-regional flow may thus reflect patients avoiding the congested journey to attend Accra’s hospitals. More generally, such inter-regional flows may affect estimates of regional skilled birth attendance rates derived from regional population counts and HMIS aggregate number of women. The attending (denominator) population served by Eastern Region’s hospitals would be underestimated, leading to an overestimate of skilled birth attendance rates in Eastern, while the opposite effect would occur in Greater Accra. To appropriately plan for such inter-regional flows, there should be a dialogue between health managers from both regions. Also, health managers and planners need to account for the true catchment of health facilities as opposed to planning within administrative boundaries not designed for healthcare delivery.

In applying study quality criteria used in a recent systematic review of healthcare attendance literature,^35^ our study based on HMIS records has several advantages over the largely community-based approaches reported to literature included in the review. First, in contrast to our study’s use of clinical records, community-based approaches generally rely on self-reported facility attendance by mothers, with potential for social desirability^36^ and recall biases.^35^ Second, in many studies included in previous systematic reviews, distance or travel time to facility is similarly self-reported, rather than calculated between origin and facility. Finally, in contrast to many of these previous studies, our analysis is based around clearly defined journey endpoints, the locations of secondary care facilities.

Studies of completeness of HMIS data in Ghana suggest the proportion of missing data is declining over time, following continued investment and data auditing.^37^ Thus, while we included a covariate to account for the proportion of missing data in our spatial interaction model, in future years, greater facility reporting may reduce the need for such measures.

The major limitation of this study is potential selection bias arising from gaps in HMIS data. Primary healthcare facilities were excluded from our analysis due to incompleteness of HMIS data, although they report a significant number of births^38^ and compete with hospitals. The routine birth data were incomplete for some hospitals and non-existent for others, which leaves gaps in the visualisation and analysis. Since more private and faith-based hospitals lacked individual-level HMIS data, such facilities are under-represented in our analysis. This means that our analysis of bypassing is based on a subgroup of hospitals where public facilities predominate and may not reflect birthing uptake at all facilities. However, the 2017 Ghana Maternal Health Survey reported only 6.3% of births in private health facilities in the Eastern Region, so this is likely to be a small proportion of births.^39^ Among those secondary care facilities that did hold individual-level records of births, the majority had fewer individual records than the aggregate total annual number of women, suggesting record incompleteness. We were unable to assess if systematic differences existed between women with and without digital records, but anecdotal evidence suggests data entry backlogs meant births later in the year were less likely to be digitised.

Incomplete geocoding of women’s places of residence may also potentially lead to a biased subset of birthing records being included in our analysis. However, there was no systematic difference in the demographics of women at places that were geocoded and those that were not (online supplementary appendix A). Furthermore, given the launch of a Ghanaian National Digital Address System and associated postal codes in 2017,^40^ in future, loss of spatial data during geocoding should reduce as this system becomes more widely used.

Furthermore, the survey data used to estimate the healthcare quality index were collected in 2010, leaving a 6-year gap between the birth data and the quality index. Due to the aggregate nature of the variables included in a classic spatial interaction model, the study could not account for individual confounders such as financial accessibility, education and perceived need for birthing services; or investigate the individual factors relating to bypassing of health facilities. While there are more realistic measures of proximity, a simple Euclidean distance measure was adopted.^24^ However, a sample of origin–destination pairs was used to compare travel time, mechanised network distance and Euclidean distance to evaluate this choice of distance metric, and this showed a high correlation between all three measures. Finally, we assumed women giving birth in hospitals should have a skilled attendant at birth. Although this might not always be the case in some primary care facilities, this assumption seems plausible given the staff complements in the region’s secondary care facilities.

## Conclusion

This study has demonstrated the utility of routine HMIS data in research to improve service provision. It shows that quality of care and distance are important influencers of choice of hospital for childbirth. Patterns of utilisation in this study could be used to inform the design of more realistic catchments for hospitals irrespective of district boundaries. Calculation of skilled birth rates per facility and district should also consider the actual rather than assumed catchment population. Given that women cross regional boundaries to attend facilities for childbirth, there should be inter-regional collaboration to better serve women on the borders of Eastern and Greater Accra regions. In future, continued investment in HMIS and the development of an address referencing system in Ghana should reduce the main limitations affecting our analysis, namely issues arising from gaps in healthcare records and incomplete or inaccurate geocoding of patients’ place of residence.
