# Supplementary material for: The influence of distance and quality on utilisation of birthing services at health facilities in Eastern Region, Ghana
Source: BMJ Glob Health. 2020 Feb 10;4(Suppl 5):e002020. doi: 10.1136/bmjgh-2019-002020 (PMC7044703; doi:10.1136/bmjgh-2019-002020)
Supplement: Supplementary data [file bmjgh-2019-002020supp001.pdf]

Supplementary material

Appendix A

The tables 1a and b below compares the characteristics of women that were successfully geocoded with women who were not. The results show similar proportions across the groups in each variable. This suggests that the women who were geocoded or not are similar. Therefore, the analysis might not be affected by systematic bias.

Table 1a: Comparison of systematic difference in background of women geocoded

		geocoded		not geocoded	
		Count	Percent	Count	Percent
Education	JHS / Middle School	12922	55.6	1361	52.9
	None	3410	14.7	451	17.5
	Primary	2257	9.7	259	10.1
	SHS / Secondary	3032	13.0	323	12.5
	Technical/Vocational	152	0.7	27	1.0
	Tertiary	1473	6.3	153	5.9
Health insurance	Not insured	255	1.1	29	1.1
	Insured	22991	98.9	2545	98.9
Outcome of delivery	Absconded	11	0.0	3	0.1
	Died	5	0.0	0	0.0
	Discharged	23205	99.8	2567	99.7
	Transferred	23	0.1	4	0.2
	Unspecified	2	0.0	0	0.0
Occupation	Employed	5018	21.6	546	21.2
	Others	4628	19.9	445	17.3
	Student	1314	5.7	123	4.8
	Trader/Farmer	10095	43.4	1162	45.1
	Unemployed	2191	9.4	298	11.6
Total deliveries	Caesarean and other surgical procedures	5506	23.7	694	27.0
	Spontaneous vaginal delivery including episiotomy	17740	76.3	1880	73.0

Table 1b: Comparison of systematic difference in background of women geocoded

		Mean	Standard Deviation	Median	Min	Max
Geocoded (n=23896)	Parity	1.67	1.40	1.00	0.00	15.00
	Age	27.38	6.45	27.00	0.00	50.00
Not geocoded (n=2633)	Parity	1.63	1.41	1.00	0.00	5.00
	Age	27.54	6.58	27.00	1.00	48.00

Appendix B:

Quality index

The quality of care index was created from the 2010 Ghana EmONC survey data. This survey sampled 1,268 health facilities nationwide from all regions, including all tiers of delivery service providers and some private health facilities. In the Eastern Region, 124 health facilities were surveyed including 22 hospitals and 52 health centres. The main inclusion criteria was facilities that had consistently recorded at least 5 births each month in 2009. Data were collected with 12 modules/questionnaires that covered infrastructure, human resources, equipment, drugs, supplies, case summary, signal functions, referral, mortalities, staff knowledge and staff technical abilities [1].

Measuring quality of maternal health delivery can be based on two themes that are provision and experience of care [2]. The provision of care is usually measured with standards and protocols outlined for service delivery whereas the experience of care focuses on aspects of service delivery that could influence the perceived quality of clients. This type of framework has been implemented to measure the quality of delivery services and report basic proportions in India [3], Nigeria [4] and Ghana [5]. Also, other measures of maternal health created summary indices from a group of variables to assess inequalities [6-8]. The bed complement of hospitals has also been included in gravity models as a proxy for the size or capacity of hospitals [9 10].

Since the objective of creating the quality index was to plug it into a gravity model, a summary measure was deemed more appropriate. Thus a composite index was created with the provision of care, the experience of care and physical size variables.
Staff strength and bed complement were selected as measures of the physical capacity of the hospitals [11]. The core staff involved in maternal health care and skilled delivery was included in the index. These group of staff are obstetrician doctors, general practitioner, general surgeon, midwives, community health nurses, medical assistant, and anaesthesiologist. The scores were normalised with range standardisation to rescale the scores between 0 and 1. Similarly, the total bed complement, maternity beds, and delivery beds were standardised as shown in Table 2.

Essential drugs used in performing specific EmONC indicators were also assessed. Oxytocin was chosen as the major uterotonic, Diazepam for anticonvulsants and antibiotics. The drugs availability questions were Yes/No types and coded as 1 for yes and 0 for no. Hence, the three variables were added up to have a total maximum score of 3.

Furthermore, medical supplies that aid in the performance of maternal health services and promotes the safety of the child were also added to the index. Ambubags were included in the index because they were essential for newborn resuscitation. Other variables in this group were partograph for monitoring of births, weighing scale to weight newborns, stethoscope to check pulse, and a cup for expressing breastmilk. These five variables were added to create one group for medical supplies.

Hospital infrastructure is an important indicator of perceived quality of care [12]. Availability of electricity, water, running water, partitioning for privacy, functioning patient latrine, and waiting area for family and friends were added to create a group for hospital infrastructure.

Infection prevention items for healthcare workers were included. Variables chosen were the availability of antiseptic, soap for handwashing, bleach for disinfection, single-use towels, Veronica bucket and a covered contaminated waste bin with a pedal.

The final group of indicators added was the EmONC indicator that included the nine signal functions. Since the objective was to create an index and not classify the health facilities by their EmONC status, the nine EmONC signal functions were summed into one variable. The

signal functions were the administration of parenteral antibiotics, administration of uterotonics, administration of parenteral anticonvulsants, manual removal of placenta, removal of retained products, assisted vaginal delivery, newborn resuscitation, blood transfusion, and performance of caesarean section. Table 2 shows the variables, score and weights used to calculate the quality of care index.

Table 2: Weighting of quality index variables

**Variable**	**Total possible score**	**Weight**
Physical size		
Staff strength<br>Total number of Obstetrician, general practitioner, general surgeon, midwife, medical assistant, community health nurse and anesthesiologists.		3
Inpatient bed complement, maternity beds, delivery beds		3
Drugs		
Oxytocin, Diazepam, Antibiotics	3	1
Medical supplies		
Ambubag, Stethoscope,<br>Partograph, weighing scale,<br>cup for breastfeeding	5	2
Non-medical supplies		
Electricity, Water, Running water,<br>Functioning patient toilet<br>Privacy, Waiting area for family	6	3
Infection prevention		
Antiseptic, Soap,<br>Veronica bucket, Bleach,<br>Single-use towel, Covered bin with pedal	6	1
EmONC		
Parenteral antibiotics, Uterotonics<br>Anticonvulsants, Manual removal of placenta,<br>Removal of retained products,<br>Assisted vaginal delivery, Newborn resuscitation<br>Blood transfusion, Caesarean section	9	2

To prevent the weights giving any group in the final index an unfair advantage, a range standardisation was applied to all the categories rescaling them from zero to one before multiplying by their weights. The normalised values were calculated as shown in Equation **3.5**.

$$\mathrm{z} = \frac{x - \min(x)}{\max(x) - \min(x)}$$

[3.5]

Where:

Z= standardised score, X = score, min(x) = minimum score for the variable, and max(x) = maximum score for the variable.

The final quality of care index was calculated using the normalised scores and weight assigned to the variables. The scores were weighted for each group of indicators, divided by the highest possible score (12) and multiplied by 100 to arrive at a percent score for each health facility. The formula used for calculating the index is shown in Equation **3.6** below:

$$Quality\ Index = \frac{3A + 3\mathrm{B} + 3\mathrm{C} + 3\mathrm{D} + 2\mathrm{E} + \mathrm{F} + 2\mathrm{G} + 3\mathrm{H} + \mathrm{I}}{12} \times 100$$

[3.6]

Where:

A = Range standardised scores of total beds

B = Range standardised scores of maternity beds

C = Range standardised scores of delivery beds

D = Range standardised scores of total staff

E = Range standardised scores of EmONC signal functions

F = Range standardised scores of infection prevention

G = Range standardised scores of medical supplies

H = Range standardised scores of non-medical supplies

I = Range standardised scores of drugs

The weights applied to the groups were subjective but guided by the Hulton, et al. [4] framework. Variables that could easily influence the perceived quality of care such as functioning patient toilet, waiting area for family and friends, privacy, electricity and water and physical size of the hospital were given a higher weight [13]. The other variables that measure the quality of care delivery with standards and protocols were weighted less since they might not be ranked higher by clients and their family.

Appendix C:

Flow diagram showing excluded data

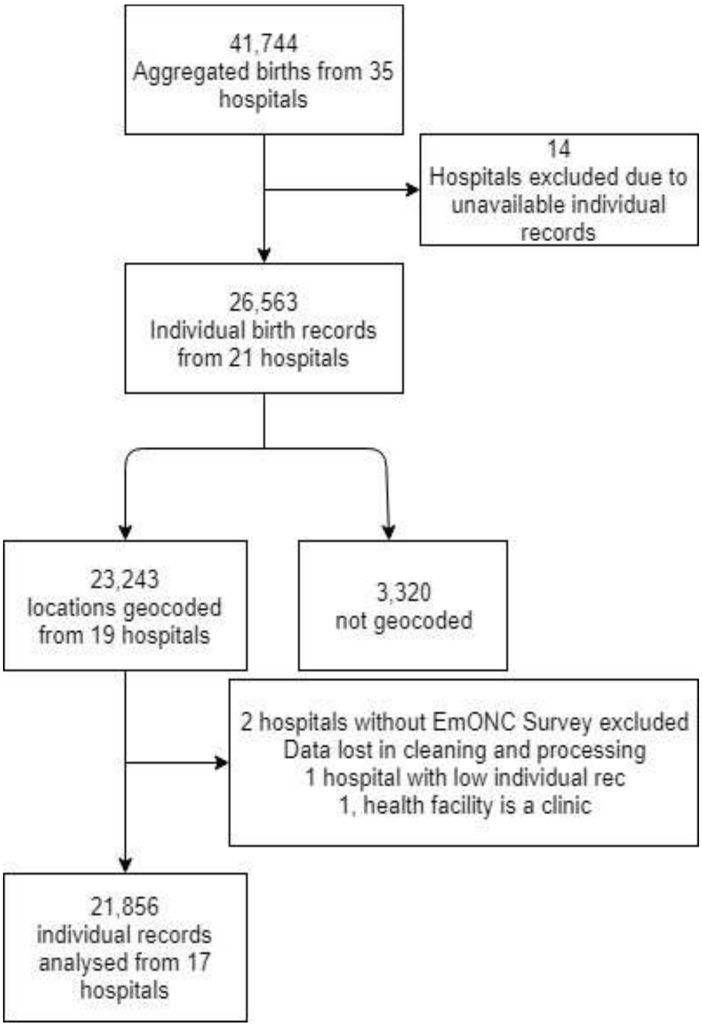

Appendix D:

Table 3: Table 3: Results from the spatial interaction model predicting logged number of journeys to give birth in 19 hospitals in Eastern Region, Ghana in 2016

Predictors	$\beta(95\%CI)$	p-value	AIC	
Poisson regression model				
Distance in Km	-0.154 (-0.156, -0.153)	<0.0001	76260.07	
Percent quality score	0.019 (0.017, 0.021)	<0.0001		
Number of estimated pregnancies	0.000 (0.0005, 0.0006)	<0.0001		
Percent completeness of deliveries data	0.016 (0.016, 0.017)	<0.0001		
Number of inpatient beds	-0.0001 (-0.0003, 0.00005)	0.154		
Zero-Inflated Poisson model				
Distance in Km	-0.088 (-0.091, -0.087)	<0.0001	59042.08	
Percent quality score	0.023 (0.021, 0.025)	<0.0001		
Number of estimated pregnancies	0.0005 (0.0005, 0.0006)	<0.0001		
Percent completeness of deliveries data	0.014 (0.013, 0.015)	<0.0001		
Number of inpatient beds	-0.001 (-0.001, -0.0008)	<0.0001		
Model inflation- Distance	0.08 (0.073, 0.084)	<0.0001		
Negative Binomial model				
Distance in Km	-0.115 (-0.120, -0.110)	<0.0001	13779.07	
Percent quality score	0.003 (-0.008, 0.015)	0.579		
Number of estimated pregnancies	0.001 (0.000, 0.001)	<0.0001		
Percent completeness of deliveries data	0.015 (0.012, 0.018)	<0.0001		
Number of inpatient beds	0.006 (0.005, 0.008)	<0.0001		
Zero-Inflated negative binomial model				
Distance in Km	-0.067 (-0.073, -0.062)	<0.0001	13456.4	
Percent quality score	0.011 (-0.001, 0.023)	0.069		
Number of estimated pregnancies	0.002 (0.001, 0.002)	<0.0001		
Percent completeness of deliveries data	0.02 (0.016, 0.024)	<0.0001		
Number of inpatient beds	0.002 (0.001, 0.004)	0.0010		
Model inflation- Distance	0.08 (0.074, 0 .087)	<0.0001		

References

1. GHS. National assessment for emergency obstetric and newborn care, Ghana, 2011.
2. Hulton L, Matthews Z, Stones RW. A framework for the evaluation of quality of care in maternity services. 2000
3. Hulton LA, Matthews Z, Stones RW. Applying a framework for assessing the quality of maternal health services in urban India. *Social Science & Medicine* 2007;64(10):208395. doi: http://dx.doi.org/10.1016/j.socscimed.2007.01.019
4. Hulton L, Matthews Z, Bandali S, et al. Accountability for quality of care: Monitoring all aspects of quality across a framework adapted for action. *International Journal of Gynecology & Obstetrics* 2016;132(1):110-16.
5. Nesbitt RC, Lohela TJ, Manu A, et al. Quality along the continuum: A health facility assessment of intrapartum and postnatal care in Ghana. *PLOS ONE* 2013;8(11):e81089. doi: 10.1371/journal.pone.0081089
6. Wehrmeister FC, Restrepo-Mendez M-C, Franca GV, et al. Summary indices for monitoring universal coverage in maternal and child health care. *Bulletin of the World Health Organization* 2016;94(12):903.
7. Barros AJD, Victora CG. Measuring coverage in MNCH: Determining and interpreting inequalities in coverage of maternal, newborn, and child health Interventions. *PLOS Medicine* 2013;10(5):e1001390. doi: 10.1371/journal.pmed.1001390
8. Hussein J, Bell J, Nazzar A, et al. The skilled attendance index: Proposal for a new measure of skilled attendance at delivery. *Reproductive Health Matters* 2004;12(24):160-70. doi: 10.1016/S0968-8080(04)24136-2
9. McGuirk MA, Porell FW. Spatial patterns of hospital utilization: the impact of distance and time. *Inquiry* 1984:84-95.
10. Congdon P. A bayesian approach to prediction using the gravity model, with an application to patient flow modeling. *Geographical Analysis* 2000;32(3):205-24. doi: 10.1111/j.1538-4632.2000.tb00425.x
11. Dogba M, Fournier P. Human resources and the quality of emergency obstetric care in developing countries: a systematic review of the literature. *Human Resources for Health* 2009;7(1):7. doi: 10.1186/1478-4491-7-7
12. Rao KD, Peters DH, Bandeen-Roche K. Towards patient-centered health services in India—a scale to measure patient perceptions of quality. *International Journal for Quality in Health Care* 2006;18(6):414-21. doi: 10.1093/intqhc/mzl049
13. Lohela TJ, Campbell OMR, Gabrysch S. Distance to care, facility delivery and early neonatal mortality in Malawi and Zambia. *PLOS ONE* 2012;7(12):e52110. doi: 10.1371/journal.pone.0052110
